# Preliminary epidemiological and clinical profile of the 2024 pediatric pertussis outbreak: a retrospective analysis

**DOI:** 10.3389/fped.2026.1816320

**Published:** 2026-06-29

**Authors:** Xiang Lin, Yuhong Qiu, Zhifang Huang, Quan Lin, Yingjie Sheng, Qingchun Zeng, Heng Tang

**Affiliations:** 1 Department of Pediatrics, Fuzhou Second General Maternity and Child Health Care Hospital, Fuzhou, China; 2 Department of Respiratory Medicine, Children’s Hospital of Nanjing Medical University, Nanjing, China

**Keywords:** clinical markers, epidemiology, pediatric infectious diseases, pertussis, pertussis resurgence

## Abstract

**Background:**

In 2024, a global surge in pediatric pertussis cases emerged, deviating from traditional clinical patterns. This outbreak was characterized by atypical symptoms and increased hospitalization rates. This study aimed to characterize the epidemiological and clinical features of children hospitalised with pertussis during the outbreak in the Fuzhou region of China and to identify predictors of prolonged hospitalization.

**Methods:**

We performed a retrospective analysis of PCR-confirmed pediatric pertussis cases admitted to Fuzhou Second General Hospital in 2024. We evaluated demographics, clinical signs, lab results, and treatment. Univariable and multivariable linear regression analyses were performed to identify factors associated with hospitalization duration. Additional LASSO regression analysis was performed as a sensitivity analysis.

**Results:**

A total of 69 children with PCR-confirmed pertussis were included in the analysis. In 2024, the pertussis outbreak began in April, peaked in June-July, and declined by August. Infants and preschool-aged children bore the highest disease burden, with a notable incidence in school-aged children. Unexpectedly, prolonged hospitalization correlated with the absence of fever and normal inflammatory markers. Multivariate analysis identified elevated platelet counts (*β* = 0.008, *p* = 0.003) and increased alanine aminotransferase (ALT) levels (*β* = 0.038, *p* = 0.018) as key predictors of extended hospital stays, offering new insights for clinical risk assessment.

**Conclusions:**

In this retrospective study of children hospitalised with pertussis during the 2024 Fuzhou outbreak, infants and school-aged children were disproportionately affected, with a seasonal distribution. Prolonged hospital stays were linked to afebrile presentations with normal inflammatory markers, while elevated platelet counts and ALT levels may predict extended hospitalization. These findings may offer useful reference points for clinical risk stratification, though validation in larger multicenter prospective studies is warranted.

## Introduction

Pertussis, an acute respiratory infection caused by *Bordetella* pertussis or *Bordetella* parapertussis, persists as a substantial public health challenge worldwide (1, 2). The disease is characterized by paroxysmal cough, whooping cough and can lead to serious complications, particularly in infants (3). While vaccination has substantially decreased pertussis incidence since its introduction in the mid-20th century, recent decades have witnessed periodic resurgences worldwide (4).

The year 2024 marked a notable increase in pertussis cases globally, with multiple countries reporting unexpected outbreaks (1, 5–7). The shifting age distribution, coupled with evolving clinical manifestations, presents new challenges for healthcare providers in diagnosis and management (8).

Understanding the factors that influence disease severity and hospital stay duration becomes crucial for optimal patient care (9). Traditional markers of disease severity may not reliably predict clinical course in pertussis infection, necessitating the identification of more accurate prognostic indicators (10). Furthermore, the clinical characteristics of pertussis in the current outbreak may differ from historical patterns, requiring updated analysis for appropriate management strategies (1).

This study aimed to describe the epidemiological trends of pertussis cases in Fuzhou during 2024 and to analyze the clinical characteristics of hospitalized children. Additionally, we sought to identify risk factors associated with prolonged hospitalization, which could aid in early recognition of cases requiring extended medical care. These findings may contribute to improved clinical decision-making and resource allocation in managing pediatric pertussis cases.

## Methods

### Study design and population

This single-center retrospective cohort study was conducted at Fuzhou Second General Hospital during the pertussis resurgence from January to August 2024. Eligible patients were those hospitalized children aged 1 month to 18 years who presented with respiratory symptoms (newborns <30 days were excluded) and had polymerase chain reaction (PCR)-confirmed pertussis infection. Children were excluded if they had chronic respiratory diseases or conditions that significantly affected immune status or cardiopulmonary function. The study protocol received approval from the institutional ethics committee, and a waiver of informed consent was granted due to the retrospective nature of the study and the use of anonymized data.

### Data collection

Data were extracted from the electronic medical record system. The following information was collected: demographic characteristics (age, gender, weight), vaccination status, clinical manifestations (whooping cough, fever, pre-hospital symptom duration), radiographic findings (bronchopneumonia), and treatment modalities (use of macrolides, steroids, sulfonamides, other antibiotics, department of hospitalization and oxygen therapy). Laboratory parameters included complete blood count with white blood cell count, lymphocyte, neutrophil, and monocyte percentages, hemoglobin, and platelet count. Inflammatory markers (C-reactive protein, CRP) and biochemical indices including alanine aminotransferase (ALT), aspartate aminotransferase (AST), lactate dehydrogenase (LDH), creatine kinase (CK), and creatine kinase-MB (CK-MB) were also collected. Laboratory parameters were categorized as elevated based on the following criteria: CRP >8 mg/L, monocyte percentage >8%, platelet count >300 × 10^9^/L, and WBC count exceeding the age-specific upper reference limits (≥6 months to <1 year: >14.6 × 10⁹/L; 1 to <2 years: >14.1 × 10⁹/L; 2 to <6 years: >11.9 × 10⁹/L; 6 to <13 years: >11.3 × 10⁹/L; 13 to 18 years: >11.0 × 10⁹/L) (11).

### Statistical analysis

Statistical analyses were performed using R software (version 4.4.1). Missing data were handled using multiple imputation with predictive mean matching method (5 imputations) to minimize potential bias. Continuous variables were presented as median [interquartile range] and compared using Mann–Whitney U test, while categorical variables were expressed as frequencies (percentages) and compared using Chi-square test or Fisher's exact test.

For the regression analysis, continuous variables were standardized to have a mean of 0 and standard deviation of 1 to facilitate comparison of effect sizes. Variables included in the multivariable model were selected based on clinical relevance and univariable analysis to avoid overfitting given the limited sample size. An additional LASSO regression analysis was performed as a sensitivity analysis. Multicollinearity was assessed using variance inflation factors (VIF), and model assumptions were verified through residual analysis. All statistical tests were two-sided, with *p* < 0.05 considered significant.

## Results

### Epidemiologic trends

**Figure 1:**
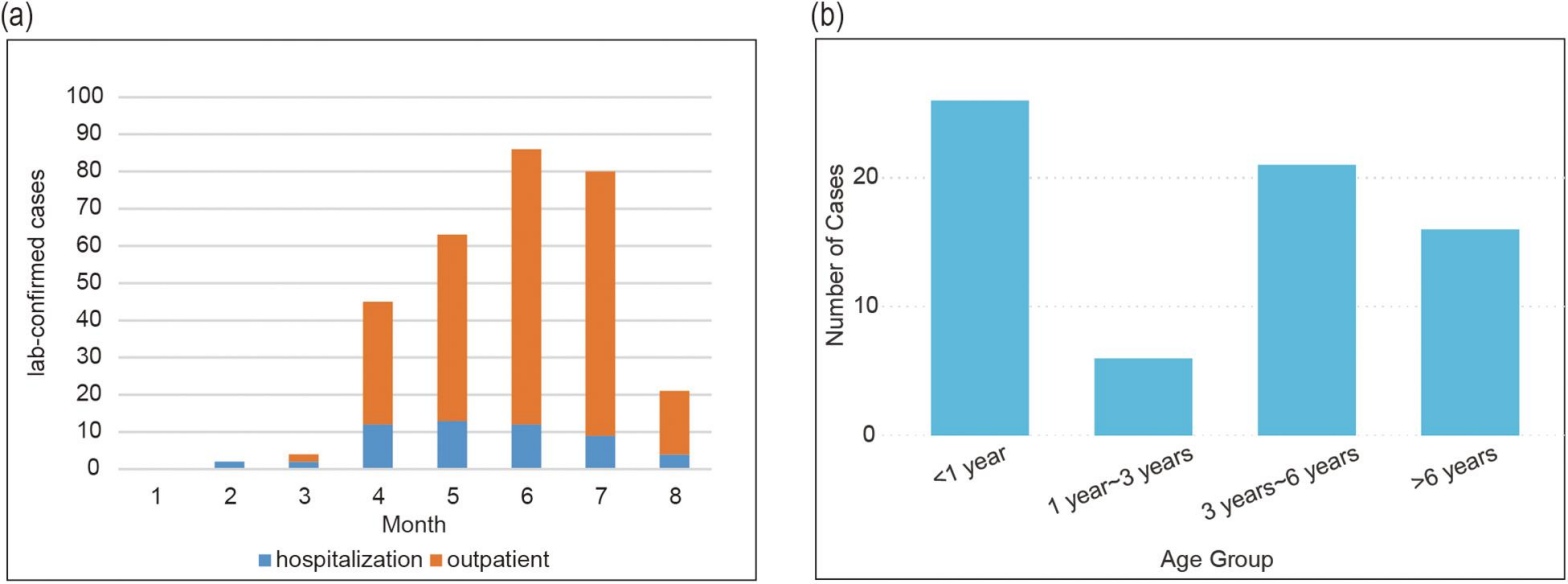
Seasonal and age-specific distribution of children with pertussis at Fuzhou Second General Hospital in 2024. **(a)** Monthly case counts showing seasonal pattern; **(b)** Age distribution of cases.

The 2024 pertussis outbreak exhibited distinct temporal and age-related patterns. The number of children with pertussis increased from April, peaked during June and July, and declined by August, indicating a clear and atypical seasonal trend (Figure 1A). Age-specific analysis (Figure 1B) revealed the highest case numbers among children younger than 1 year, accounting for approximately 35% of all cases, followed by those aged 3–6 years.

### Study population characteristics

A total of 73 children with PCR-confirmed pertussis were initially identified. After excluding 3 patients with missing key clinical information and 1 patient with severe comorbidities, 69 patients remained for the final analysis (Table 1). The median age of the cohort was 4.00 years (IQR, 0.33–6.00 years), and 60.9% (42/69) of the children were male. The median duration of pre-hospital symptoms was 7.00 days (IQR, 6.00–14.00 days). Overall, 64 patients (92.8%) were vaccinated against pertussis.

**Table 1:** Baseline characteristics of patients with Pertussis stratified by length of hospital stay.

Characteristic	Overall	LOS < 8 days	LOS ≥ 8 days	*p*-value
Demographics				
n	69	42	27	-
Age, median [IQR], years	4.00 [0.33, 6.00]	4.00 [0.85, 6.00]	4.00 [0.25, 7.00]	0.626
Gender, male, *n* (%)	42 (60.9)	24 (57.1)	18 (66.7)	0.590
Weight, median [IQR], kg	15.00 [7.50, 21.00]	7.50 [6.00, 11.50]	7.00 [7.00, 14.00]	0.499
Pre-Hospital symptom duration, median [IQR], days	7.00 [6.00, 14.00]	15.00 [9.00, 20.00]	16.00 [6.00, 22.00]	0.754
Vaccination Status=Vaccinated	64 (92.8)	41 (97.6)	23 (85.2)	0.142
Symptoms				
Cough, *n* (%)	68 (98.6)	41 (97.6)	27 (100.0)	1.000
Fever, *n* (%)	24 (34.8)	18 (42.9)	6 (22.2)	0.134
Radiography finding				
Bronchopneumonia, *n* (%)	27 (42.2)	19 (45.2)	13 (48.1)	1.000
Laboratory Tests				
CRP, median [IQR], mg/L	0.84 [0.50, 6.27]	0.95 [0.50, 6.27]	0.50 [0.50, 2.82]	0.243
WBC, median [IQR], ×10^9^/L	11.70 [8.80, 16.50]	10.35 [8.13, 15.10]	15.70 [10.85, 18.00]	0.014
Lymphocyte %, median [IQR]	48.60 [34.75, 66.35]	44.30 [29.75, 64.27]	53.70 [36.20, 70.50]	0.285
Neutrophil %, median [IQR]	41.70 [22.45, 55.85]	46.20 [25.65, 60.38]	27.90 [21.80, 52.55]	0.240
Monocyte %, median [IQR]	5.90 [4.70, 7.10]	6.25 [4.80, 7.57]	5.40 [4.25, 6.10]	0.020
Hemoglobin, median [IQR], g/L	123.00 [116.00, 131.50]	124.00 [118.00, 132.00]	120.00 [111.00, 133.00]	0.510
Platelet, median [IQR], ×10^9^/L	370.00 [296.50, 447.50]	339.50 [294.75, 394.25]	440.00 [357.00, 515.50]	0.005
ALT, median [IQR], U/L	16.40 [11.30, 26.70]	14.75 [10.60, 23.23]	20.80 [12.85, 29.65]	0.091
AST, median [IQR], U/L	31.55 [26.10, 42.55]	32.80 [27.65, 42.65]	30.40 [20.95, 38.90]	0.228
LDH, median [IQR], U/L	279.75 [241.17, 329.98]	282.05 [249.02, 331.92]	271.60 [233.05, 320.80]	0.680
CK, median [IQR], U/L	121.10 [82.72, 172.00]	121.10 [84.70, 204.93]	102.60 [71.50, 156.45]	0.439
CK-MB, median [IQR], U/L	29.30 [19.32, 38.62]	30.20 [21.30, 41.38]	29.20 [18.15, 33.60]	0.307
Treatment				
Steroid Use, *n* (%)	42 (60.9)	26 (61.9)	16 (59.3)	1.000
Macrolide Use, *n* (%)	54 (78.3)	32 (76.2)	22 (81.5)	0.825
Sulfonamide Use, *n* (%)	10 (14.5)	6 (14.3)	4 (14.8)	1.000
Other Antibiotic Use, *n* (%)	18 (26.1)	8 (19.0)	10 (37.0)	0.168
Oxygen Therapy, *n* (%)	3 (4.3)	0 (0.0)	3 (11.1)	0.109
Outcome				
LOS, median [IQR], days	7.00 [6.00, 8.00]	6.00 [4.00, 7.00]	9.00 [8.00, 10.00]	-

LOS, length of hospital stay (days); CRP, C-reactive protein (mg/L); WBC, white blood cell count (×10⁹/L); PLT, platelet count (×10⁹/L); ALT, alanine aminotransferase (U/L); AST, aspartate aminotransferase (U/L); LDH, lactate dehydrogenase (U/L); CK, creatine kinase (U/L); CK-MB, creatine kinase MB isoenzyme (U/L).

Cough was the predominant symptom, present in 98.6% (68/69) of children, while fever was reported in 34.8% (24/69). Radiographic findings showed bronchopneumonia in 42.2% (27/69) of cases.

Laboratory test results revealed a median WBC count of 11.70 × 10^9^/L (IQR, 8.80–16.50 × 10^9^/L), with a lymphocyte percentage of 48.60% (IQR, 34.75–66.35%). CRP levels were generally low, with a median of 0.84 mg/L (IQR, 0.50–6.27 mg/L). Platelet counts were elevated in many cases, with a median of 370.00 × 10^9^/L (IQR, 296.50–447.50 × 10^9^/L), while hemoglobin levels were within the normal range (median, 123.00 g/L; IQR, 116.00–131.50 g/L). Both ALT and AST levels remained within the normal range in both groups.

Regarding treatment, macrolide antibiotics were administered to 78.3% (54/69) of children, and sulfonamides to 14.5% (10/69). Corticosteroids were used in 60.9% (42/69) of cases, and oxygen therapy was required in 4.3% (3/69).

### Prolonged hospital stay linked to higher WBC, PLT, and lower monocyte percentage

Using the median length of stay (7 days) as a reference, as shown in Table 1, patients were stratified into two groups: prolonged hospital stay (≥8 days, *n* = 27) and normal stay (<8 days, *n* = 42). Several significant differences emerged in laboratory parameters between these groups. Patients with prolonged hospital stay demonstrated significantly higher white blood cell counts (15.70 vs. 10.35 × 10^9^/L, *p* = 0.014) and platelet counts (440.00 vs. 339.50 × 10^9^/L, *p* = 0.005). Interestingly, the monocyte percentage was significantly lower in the prolonged stay group (5.40% vs. 6.25%, *p* = 0.020). Vaccination status did not differ significantly between the shorter-stay and prolonged-stay groups (97.6% vs. 85.2%, *p* = 0.142).

### Infant age and atypical inflammatory response associated with Longer hospitalization

**Figure 2:**
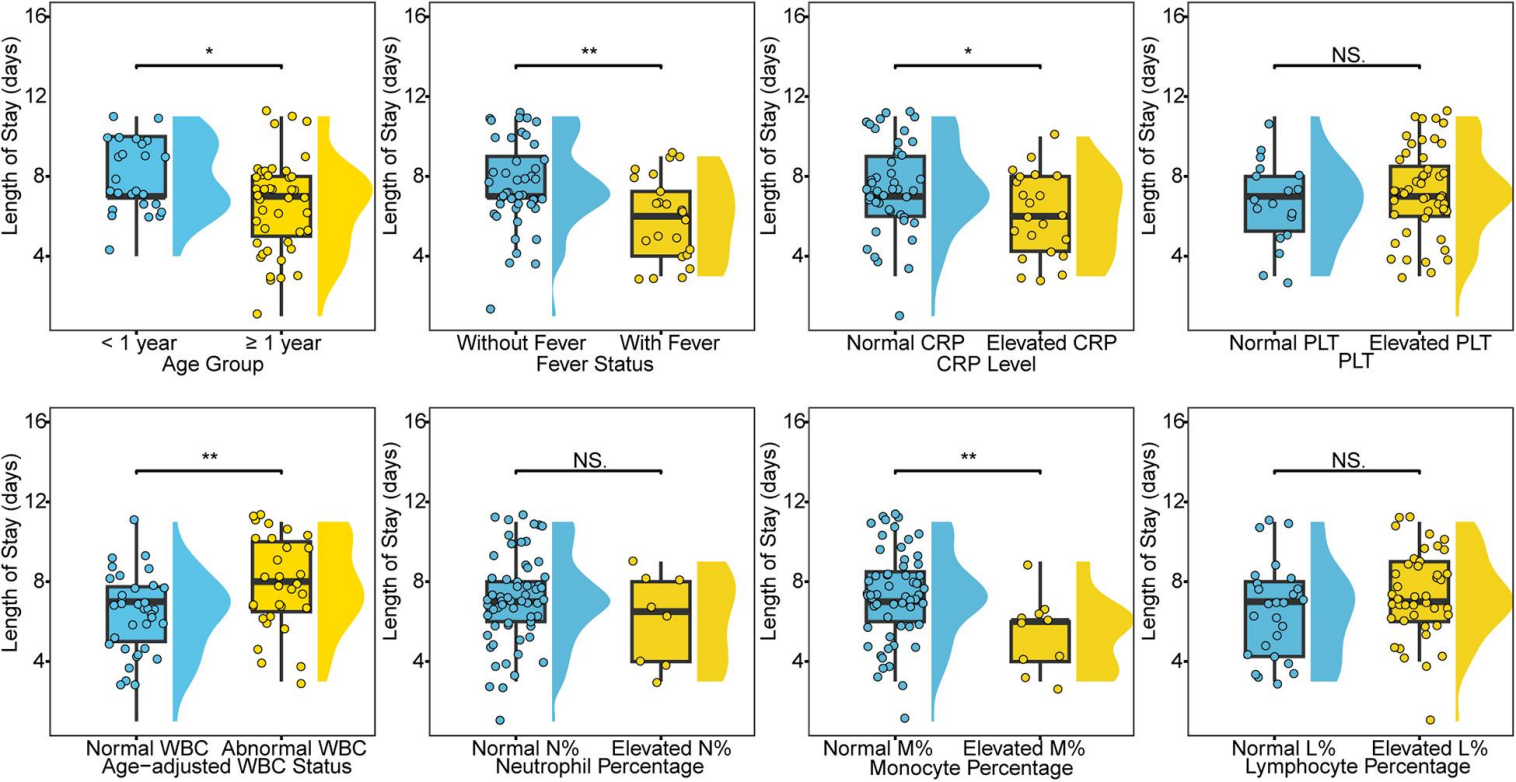
Association of clinical and laboratory factors with hospital stay duration in pediatric Pertussis. LOS, length of hospital stay (days); CRP, C-reactive protein (mg/L); WBC, white blood cell count (×10⁹/L); PLT, platelet count (×10⁹/L); N, neutrophils; M, monocytes; L, lymphocytes.

Analysis of clinical factors revealed several noteworthy patterns in hospital stay duration (Figure 2). Demographically, infants under one year of age had a significantly longer hospital stay (median 7 days, IQR 7–10) compared to older children (median 7 days, IQR 5–8), with a median difference of 2.0 days (95% CI: 0.00008–3.0; *p* = 0.019), consistent with the known vulnerability of this age group to severe pertussis infection. Interestingly, the absence of typical inflammatory manifestations was associated with prolonged hospitalization: patients without fever had significantly longer hospital stays than those with fever (*p* < 0.01), and those with normal CRP levels had extended hospitalizations compared to those with elevated CRP (*p* < 0.05).

Among laboratory parameters, elevated WBC counts were associated with extended hospitalization (*p* < 0.05), while normal monocyte percentages, rather than elevated levels, were associated with significantly longer hospital stays (*p* < 0.01). This paradoxical relationship between conventional inflammatory markers and length of stay highlights the unique immunological characteristics of pertussis infection. No significant differences in length of stay were observed for platelet counts, neutrophil percentage, or lymphocyte percentage.

### Platelet count and ALT levels predict prolonged hospital stay

Univariable regression analyses are presented in Supplementary Table S1. Variables significantly associated with length of hospital stay (LOS) in univariable analysis, including platelet count, ALT, and fever, were entered into the multivariable model. Age was additionally included because of its established clinical relevance in pertussis. Monocyte, Neutrophil and AST were excluded due to substantial multicollinearity. Vaccination status was not included because vaccination eligibility in China is age-dependent, leading to substantial collinearity between vaccination status and age.

In the multivariable linear regression model (Table 2), higher platelet count and ALT levels were independently associated with prolonged LOS. Elevated platelet count showed the strongest association with extended hospitalization (*β* = 0.008, 95% CI: 0.003–0.013, *p* = 0.003), followed by increased ALT levels (*β* = 0.038, 95% CI: 0.002–0.075, *p* = 0.040). Age showed a trend toward positive association with hospital stay (*β* = 0.168, 95% CI: −0.010 to 0.347, *p* = 0.063), while the presence of fever demonstrated a trend toward shorter hospitalization periods (*β* = −1.020, 95% CI: −2.100 to 0.058, *p* = 0.063), though neither reached statistical significance. As a sensitivity analysis, LASSO regression was additionally performed using clinically relevant candidate variables (Supplementary Table S2). Platelet count and ALT remained retained in the penalized regression model, supporting the robustness of the primary multivariable analysis.

**Table 2:** Multiple linear regression analysis identifying predictors of hospital stay duration in patients with Pertussis .

Variable	Coefficient	SE	*t*-value	*P*-value	95% CI Lower	95% CI Upper	Significance
Age	0.168	0.089	1.86	0.063	−0.01	0.347	
PLT	0.008	0.003	3.10	0.003	0.003	0.013	**
ALT	0.038	0.018	2.10	0.04	0.002	0.075	*
Fever	−1.02	0.541	−1.889	0.063	−2.088	0.058	

* *p* < 0.05,

** *p* < 0.01.

PLT, platelet count (×10⁹/L); ALT, alanine aminotransferase (U/L); SE, standard error; CI, confidence interval.

## Discussion

This single-center retrospective cohort study provides a comprehensive analysis of the epidemiological characteristics and clinical predictors of disease severity among 69 PCR-confirmed pediatric pertussis cases during the 2024 resurgence, integrating detailed clinical, laboratory, and demographic data to identify novel patterns in disease presentation and progression.

Our retrospective analysis of 69 hospitalized children with laboratory-confirmed pertussis in Fuzhou during 2024 revealed significant epidemiological characteristics. The data demonstrated a clear summer predominance, particularly during June and July, which represents a distinct seasonal pattern compared to traditional spring or winter peaks reported in previous literature (12, 13).The demographic analysis highlighted two vulnerable populations: infants below 12 months, especially infants <3 months, comprising approximately one-third of cases, and children between 3 and 6 years (14). This bimodal age distribution likely reflects the complex interplay between vaccination timing and immunity duration (5, 15). Notably, the increased proportion of school-age children (>6 years) than historically reported in our study mirrors a global trend observed during the 2024 resurgence (6, 14, 16–18). The timing of this outbreak coincides with a broader resurgence of pertussis cases globally, which may be attributed to several factors: enhanced detection capabilities through improved diagnostic methods, diminishing vaccine-induced immunity, and the emergence of susceptible populations following periods of reduced disease transmission (5, 15). This pattern underscores the dynamic nature of pertussis epidemiology and the need for continued surveillance and adaptation of prevention strategies (13).

The analysis of factors influencing hospitalization duration yielded several intriguing findings that challenge conventional clinical expectations. Consistent with our demographic findings showing infants under one year as a major affected group, this vulnerable population also demonstrated significantly prolonged hospital stays (19). This finding is clinically plausible, as young infants, particularly those younger than 6 months, are prone to paroxysmal cough, apnea or cyanosis, post-tussive vomiting, and feeding difficulties, which may require close monitoring and supportive care despite minimal systemic inflammation (12, 20). A particularly noteworthy observation was the inverse relationship between traditional inflammatory markers and hospitalization duration. Patients without fever and those maintaining normal CRP levels paradoxically required longer hospital stays, contrasting with typical patterns seen in other respiratory infections (21). This phenomenon might reflect the unique immunopathogenesis of B. pertussis, which induces disease primarily through toxin-mediated damage rather than a robust systemic inflammatory response (21, 22). Unlike many other respiratory infections, where heightened inflammation is associated with disease severity, *B. pertussis* modulates immune pathways, leading to an atypical clinical presentation characterized by prolonged paroxysmal cough in the absence of fever or significant inflammatory markers (23, 24). The absence of strong inflammatory manifestations may reflect bacterial immune modulation rather than a functionally suboptimal immune response. These findings highlight the complex interplay between immune evasion, bacterial persistence, and clinical outcomes in pertussis, emphasizing the need for further research into immunological markers that predict disease severity and recovery (23, 25).

To further elucidate the determinants of hospital stay duration, our multivariate analysis revealed several significant predictors. Elevated platelet count emerged as the strongest independent predictor of extended hospital stays, followed by increased ALT levels. Thrombocytosis in pertussis patients may correlate with disease severity, though its underlying mechanism remains unclear. Rather than directly reflecting systemic inflammation, it may result from hypoxia-induced platelet production or cytokine-mediated thrombopoiesis (21, 22). Further research is needed to determine its clinical significance. Elevated ALT as a predictor of prolonged hospitalization may reflect secondary hepatocellular stress. Although ALT elevation has not been well characterized as a typical feature of pertussis, abnormal liver function has been associated with severe pertussis in infants in previous studies. Severe paroxysmal coughing with apnea or hypoxemia, systemic illness, reduced feeding or dehydration, and treatment-related drug exposure may contribute to transient aminotransferase elevation. In this context, elevated ALT may serve as an indirect marker of a more complicated clinical course, requiring closer monitoring and potentially delaying discharge (20, 26). While age and the absence of fever showed trends toward longer hospitalization, these associations did not reach statistical significance, consistent with *B. pertussis*'s ability to cause severe disease without robust systemic inflammation (27).

The strengths of our study include its timeliness in analyzing the current outbreak, comprehensive clinical and laboratory data collection, and the identification of practical prognostic indicators for clinical management. However, several limitations should be acknowledged. First, the single-center design restricts the generalizability of our findings, and the modest sample size may limit the statistical power of the multiple linear regression analysis. Second, the retrospective nature of the study precluded the collection of some potentially relevant data. Future multi-center prospective studies with larger cohorts, particularly those spanning multiple provinces and regions, are warranted to validate these findings.

In conclusion, this study provides valuable insights into the clinical characteristics and risk factors for prolonged hospitalization in pediatric pertussis cases during the 2024 resurgence. Our findings highlight the changing epidemiology of pertussis, particularly the increased involvement of school-age children, and identify novel predictors of extended hospital stays.

## Data Availability

The raw data supporting the conclusions of this article will be made available by the authors, without undue reservation.
