# Supplementary material for: Preliminary epidemiological and clinical profile of the 2024 pediatric pertussis outbreak: a retrospective analysis
Source: Front Pediatr. 2026 Jun 29;14:1816320. doi: 10.3389/fped.2026.1816320 (PMC13357190; doi:10.3389/fped.2026.1816320)
Supplement: Supplementary file 1 [file Table1.docx]

**Supplementary Materials**

**Manuscript Title:** Epidemiological and Clinical Profile of the 2024 Pediatric Pertussis Outbreak: A Retrospective Analysis

**Table S1.** Univariable Linear Regression Analysis Identifying Predictors of Hospital Stay Duration in Patients with Pertussis

| **Variable** | **β (unstandardized)** | **95%CI** | **p-value** |
| --- | --- | --- | --- |
| PLT | 0.008 | 0.003-0.013 | 0.0010 |
| Fever | -1.683 | -2.77--0.597 | 0.0029 |
| ALT | 0.040 | 0.004-0.075 | 0.0284 |
| Monocyte (%) | -0.289 | -0.547--0.03 | 0.0291 |
| Neutrophil (%) | -0.028 | -0.054--0.001 | 0.0416 |
| WBC | 0.074 | -0.016-0.164 | 0.1033 |
| Lymphocyte (%) | 0.008 | -0.006-0.022 | 0.2740 |
| LDH | 0.004 | -0.004-0.012 | 0.3205 |
| CK | -0.001 | -0.002-0.001 | 0.4606 |
| Age | -0.058 | -0.216-0.101 | 0.4699 |
| CRP | -0.014 | -0.062-0.034 | 0.5520 |
| Bronchopneumonia | 0.280 | -0.851-1.412 | 0.6224 |
| Pre-hospital Days | 0.020 | -0.065-0.105 | 0.6429 |
| CK-MB | -0.002 | -0.035-0.03 | 0.8999 |

**Table S2.** LASSO Regression Coefficients for Predicting Length of Stay (LOS)

| Variable | Coefficient |
| --- | --- |
| PLT (10^9/L) | 0.5001 |
| ALT (U/L) | 0.1575 |

Continuous variables were standardized (z-score). Lasso penalty λ was selected by 10-fold cross-validation (λ.min).
